# Next‐generation sequencing assisted diagnosis of cervical metastasis in *EGFR*‐mutated lung adenocarcinoma: A case report

**DOI:** 10.1111/1759-7714.14143

**Published:** 2021-09-09

**Authors:** Li Xu, Kang Li, Xiaoyan Chen, Bolin Chen, Jia Li, Lin Wu

**Affiliations:** ^1^ Second Department of Thoracic Medicine, Hunan Cancer Hospital, The Affiliated Cancer Hospital of Xiangya School of Medicine Central South University Changsha China; ^2^ Clinical Pathology Diagnostic Center, Hunan Cancer Hospital, The Affiliated Cancer Hospital of Xiangya School of Medicine Central South University Changsha China

**Keywords:** *EGFR* mutation, metastasis, next‐generation sequencing, non‐small cell lung cancer, uterine cervix

## Abstract

*EGFR* mutation has been detected in more than half of non‐small cell lung cancer (NSCLC) patients in Asia. Lung cancer is the main cause of malignant tumor‐related death worldwide. Although distant metastases often occurs in patients with advanced NSCLC, uterine cervical metastasis is rare. Here, we report a case of *EGFR*‐mutated lung adenocarcinoma with cervical metastasis. A 63‐year‐old female with known lung adenocarcinoma was found to have abnormal vaginal bleeding during osimertinib follow‐up visits. Immunohistochemical (IHC) staining and next‐generation sequencing (NGS) of the biopsy sample from the uterine cervical tumor confirmed metastatic dissemination from the primary lung malignancy. NGS assisted the diagnosis of uterine cervical metastasis from the primary lung. This is another major clinical application of NGS in addition to medication guidance and identification of drug resistance mechanisms.

## Graphical abstract

Non‐small cell lung cancer (NSCLC) with uterine cervical metastasis is rarely reported. IHC staining and NGS assisted the diagnosis of uterine cervical metastasis from the primary lung cancer.

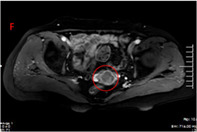

## INTRODUCTION

Metastatic spread from lung carcinoma is quite predictable, initially through lymphatic vessels followed by the hematogenous route. Common metastatic sites of lung cancer are the liver, brain, bones and adrenal glands.^1^, ^2^ However, metastasis to the uterine cervix is extremely rare. Here, we report a unique case of uterine cervical metastasis in non‐small cell lung cancer (NSCLC).

## CASE REPORT

**FIGURE 1:**
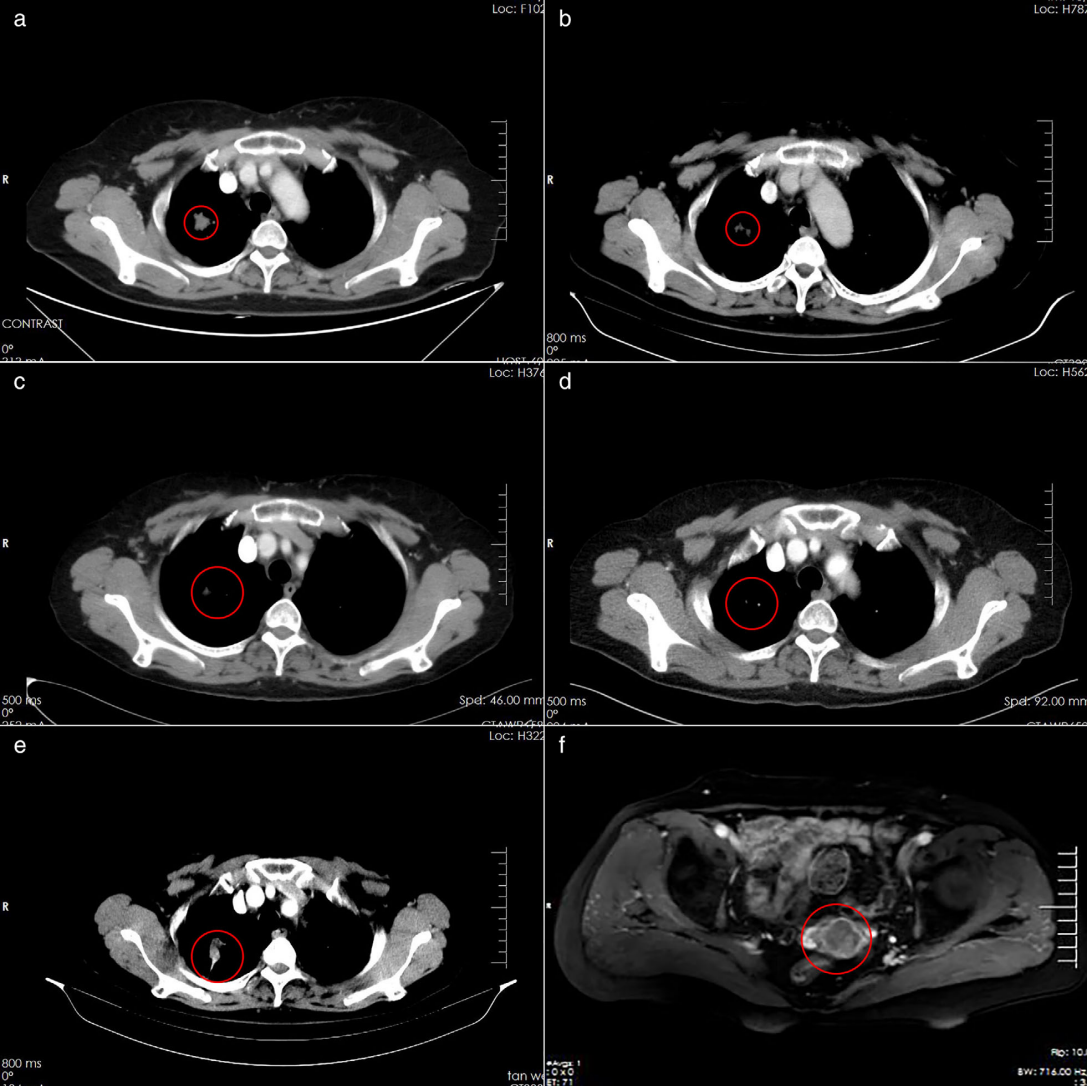
(a) and (b) Imaging before and after gefitinib treatment (a) on March 5, 2016, and (b) April 23, 2016. (c) and (d) Imaging before and after treatment with osimertinib (c) on March 22, 2018 and (d) on June 25, 2018. (e) Chest computed tomography (CT) scan on March 22, 2020. (f) Pelvic scan on April 26, 2020

**FIGURE 2:**
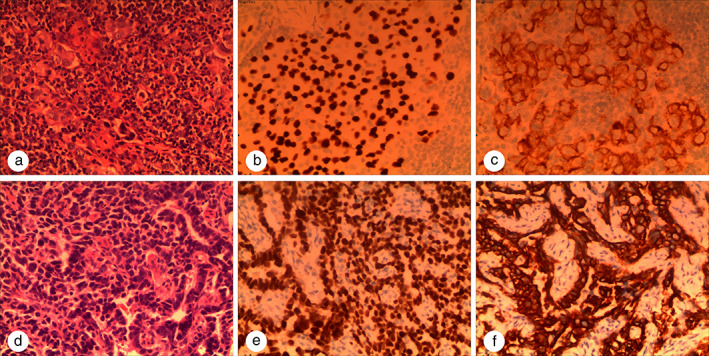
Hematoxylin‐eosin (HE) and immunohistochemical staining (×200). (a) HE staining of the biopsy sample from the right supraclavicular node, poorly differentiated adenocarcinoma. (b) and (c) Immunohistochemical staining of (b) thyroid transcription factor‐1 (TTF‐1) and (c) cytokeratin 7 (CK7) with the right supraclavicular node biopsy sample. (d) HE staining of the biopsy sample from the cervical tumor, poorly differentiated adenocarcinoma. (e and f) Immunohistochemical staining of (e) TTF‐1 and (f) CK7 with the cervical tumor biopsy sample

A 63‐year‐old woman without a smoking history presented with cough lasting for one month. Computed tomography (CT) of the thorax showed a nodule in the right upper lobe of the lung, measuring 1.5 × 2 cm (Figure 1(a)). Isotope bone scan was performed which indicated the presence of a metastatic bone lesion (Figure SS1(a)). A biopsy sample of the right supraclavicular node mass was suggestive of a poorly differentiated adenocarcinoma (Figure 2(a)). Immunohistochemical (IHC) positive staining of thyroid transcription factor‐1 (TTF‐1, Figure 2(b)) and cytokeratin 7 (CK7, Figure 2(c)) supported the diagnosis of supraclavicular node metastasis from lung cancer.

Finally, a diagnosis of IVa stage (T1N3M1b) adenocarcinoma lung cancer with bone metastasis was made. Amplification refractory mutation system (ARMS) testing showed an epidermal growth factor receptor (EGFR) exon 21 p.L858R mutation. The patient commenced gefitinib treatment in March 2016. One month later, a partial response had been achieved according to the CT scan (Figure 1(b)). The patient continued gefitinib treatment for a further 2 years until several new bone metastasis were detected on isotope bone scan in March 2018 (Figure S1(b)). The patient had accompanying lumbar vertebra pain. CT of the thorax showed the nodule remained stable in the right lung (Figure 1(c)). Quantified paired plasma and leukocyte samples from the patient were sequenced by targeted NGS with a specific 122‐gene panel. The captured samples were then subjected to Illumina HiSeq X‐Ten for paired‐end sequencing and information of gene features were obtained from Genecast Biotechnology. NGS revealed the appearance of *EGFR* exon 20 p.T790M mutation (p.T790 M) from cell free DNA in plasma. Osimertinib treatment was commenced and her pain quickly subsided. Three months later, a partial response had been achieved according to the CT scan (Figure 1(d)). Osimertinib treatment was continued for another 2 years and then in April 2020, the patient again experienced lumbar vertebra pain. Abnormal vaginal bleeding and discharge were also observed at this time. CT scan of the thorax showed a larger nodule in the upper lobe (Figure 1(e)), and magnetic resonance imaging (MRI) of the pelvis showed a mass in the uterine cervix (Figure 1(f)).

A biopsy sample was obtained from the uterine cervical mass. Hematoxylin‐eosin (HE) staining of the uterine cervical biopsy sample identified an invasive poorly differentiated adenocarcinoma (Figure 2(d)). IHC staining was strongly positive for TTF‐1 and CK7 (Figure 2(e)–(f)), while p63, CK20 were negative. The results suggested that the uterine cervical mass was metastatic adenocarcinoma of the lung. In addition, using formalin‐fixed paraffin‐embedded tissues, 15 slides with 5 μm sliced tissues were prepared for NGS analysis. Meanwhile, quantified paired plasma and leukocyte samples from the patient were sequenced by NGS with the same panel again. *EGFR* exon 21 p.L858R mutation was again detected which further verified that the uterine cervical tumor was a metastasis from primary lung adenocarcinoma. The detailed mutation results detected by NGS are described in Table 1.

**TABLE 1:** The results of next‐generation sequencing (NGS)

Mutation type	cfDNA in 2018	cfDNA in 2020	DNA from cervical biopsy in 2020
EGFR L858R	1.15%	23.32%	29.05%
EGFR T790M	0.32%	‐	‐
EGFR G242fs	0.61%	‐	‐
TP53 H179Q	0.8%	35.23%	63.37%
ALK G927fs	0.47%	‐	‐
APC P2048fs	0.81%	‐	‐
AR Q58L	11.34%	‐	‐
AR Q59L	3.12%	‐	‐
ASXL1 T707fs	1.13%	‐	‐
BRCA2 Q861fs	0.67%	‐	‐
CARD11 R555fs	0.79%	‐	‐
CIC S1595fs	1.31%	‐	‐
FGFR4 P400fs	0.7%	‐	‐
FLT3 R773fs	0.62%	‐	‐
GNAS A38fs	0.56%	‐	‐
JAK3 Q39fs	0.57%	‐	‐
KMT2C K822fs	0.59%	‐	‐
MEN1 R521fs	0.65%	‐	‐
PAX5 F27fs	0.55%	‐	‐
PDGFRA F808fs	0.55%	‐	‐
PMS2 M312V	1.16%	‐	‐
ROS1 N1821fs	0.88%	‐	‐
SETD2 T2388fs	0.73%	‐	‐
PIK3CA E545K	‐	4.51%	0.67%
PIK3CA E542K	‐	0.36%	4.03%
RB1 L586*fs*1	‐	17.72%	43.65%
NOTCH4 Amplification	‐	‐	5.71

*represents the termination codon.

## DISCUSSION

The initial tumor site of the new cervical mass was difficult to diagnose since metastasis of the feminine genital tract from noncontiguous sites is very rare. Lung metastasis to the uterine cervix is even more uncommon. The reason why metastatic carcinoma to the cervix alone through hematogenous or lymphatic spread is so rare is because of its small size, relatively limited blood flow, distal circulation, as well as organ's abundant content of fibrous tissue, which makes the uterine cervix a medium that is scarcely favorable for the propagation of malignant cells.^3^ In this case, gynecological symptoms of vaginal bleeding, pelvic pain, mass, and vaginal discharge were present. Gynecological symptoms that follow a medical history of lung carcinoma should raise a suspicion of metastases in order to rapidly refer patients for the appropriate treatment.

To determine whether the uterine cervical tumor was primary or metastasis, pathological and genomic testing were performed in this case. TTF‐1 and CK7 were positive which suggested metastatic adenocarcinoma of the lung. The NGS results of cervical cancer specimens harbored *EGFR* exon 21 p.L858R mutation which were consistent with the primary tumor tissue of the right supraclavicular node before initial treatment. Both results supported the conclusion that the uterine cervical tumor was metastasis from the lung carcinoma.

Patients with sensitive *EGFR* mutations are highly sensitive to EGFR‐TKIs. In this report, the patient had received gefitinib as first‐line treatment for 2 years, until disease progression and p.T790M mutation was tested in the plasma by NGS. p.T790M is a main reason for acquired resistance to first generation EGFR‐TKIs which abrogates the inhibitory activity of tyrosine kinase inhibitors (TKIs).^4^ It has been reported that osimertinib has significantly greater efficacy than chemotherapy in advanced NSCLC patients with p.T790M mutation in whom disease has progressed during first‐line EGFR TKI treatment.^5^, ^6^ Osimertinib was commenced in the patient in our report for a further two years. However, disease progression appeared again 2 years later with the uterine cervical metastasis. In addition to *EGFR* mutation, *PIK3CA* mutation was found both in the plasma and uterine cervical mass. It has previously been reported that *PIK3CA* mutation with loss of p.T790M mutation is a resistance mechanism for osimertinib.^7^ In this case, NGS testing not only assisted the diagnosis with the uterine cervical metastasis, but also identified the resistance mechanism of EGFR‐TKIs and guided the subsequent therapy.

*EGFR* mutations define an important molecular subtype of NSCLC. *EGFR* positive NSCLC tends to be present in patients with no smoking habit, Asian women, and adenocarcinoma. Six patients with metastatic activity to the uterus with *EGFR* mutation have previously been reported (Table 2).^8^, ^9^, ^10^, ^11^, ^12^ All had lung adenocarcinoma and were Asian women. It has also been reported that *EGFR* mutations are associated with distant metastasis, especially brain metastasis.^13^, ^14^, ^15^ *EGFR*‐mutated NSCLC may be associated with more aggressive tumor progression.^13^ Therefore, *EGFR* mutation may promote the reproductive system metastasis of female lung adenocarcinoma patients. It has also previously been reported that different factors are associated with different distant metastasis in *EGFR*‐mutated NSCLC.^16^ *EGFR* exon 19 deletion has been reported to be more associated with lung and brain metastasis, and *EGFR* exon 21 p.L858R mutation more associated with liver metastasis.^14^ Uterine cervical metastasis in *EGFR*‐mutated NSCLC may be associated with specific factors but a greater number of samples are needed to verify this.

**TABLE 2:** Previous cases of uterine metastasis of lung adenocarcinoma with *EGFR* mutation

Author/year	Age	Metastatic site	Nationality	Stage when first diagnosed	Time to diagnosis of metastatic site	Sign	Histological finding	*EGFR* mutation of LC	*EGFR* mutation of UM	Treatment after metastatic lesion diagnosis
Yong et al. 2020^8^	49	Uterine cervix	Chinese	IVb	Same time	Lumbago and sacroiliac joint pain; vaginal bleeding	TTF‐1, Napsin A, CK7 and Ki‐67(50%) positive	19del	19del	Osimertinib
Yan et al. 2019^9^	41	cervix	Chinese	IV	Same time	Frequent micturition and hypogastralgia	TTF‐1 and CK‐7 positve	Inadequate sample	L858R	Gefitinib
	29	ovary	Chinese	IV	4.5 months after icotinib initiation	Pelvic effusion	TTF‐1, CK‐7, CK‐20 and Ki67(15%) positve	19del	19del + T790M	Osimertinib
Ahmad et al. 2015^10^	51	Endometrial	Chinese	IV	22 months after initial diagnosis	Abdominal pain and heavy vaginal bleeding	TTF‐1 positive	L858R	L858R + T790M	–
Kajimoto et al. 2015^11^	82	Endometrial	Japanese	IV	Same time	Abnormal genital bleeding	‐	L858R	L858R	Death without treatment
Shibata et al. 2018^12^	63	Myometrium adjacent to myoma	Japanese	IIIB	24 months after initial diagnosis	Vaginal bleeding	TTF‐1 and Napsin A positive	19del	19del + T790M	TAH + BSO

In conclusion, although a rare occurrence, uterine cervical metastasis should be considered if patients with primary lung adenocarcinoma experience abnormal vaginal bleeding. NGS testing was of great assistance in the case reported here and for the diagnosis of rare metastatic tumors, and is a pivotal complement for immunohistochemistry.

## CONFLICT OF INTEREST

The authors report no conflict of interest.

## Supporting information

**Figure S1** Isotope bone scan at March 22th, 2016 (A) and March 21th, 2018 (B).
