# Supplementary figures and images for: Next‐generation sequencing assisted diagnosis of cervical metastasis in EGFR‐mutated lung adenocarcinoma: A case report
Source: Thorac Cancer. 2021 Sep 9;12(19):2622–7. doi: 10.1111/1759-7714.14143 (PMC8487810; doi:10.1111/1759-7714.14143)

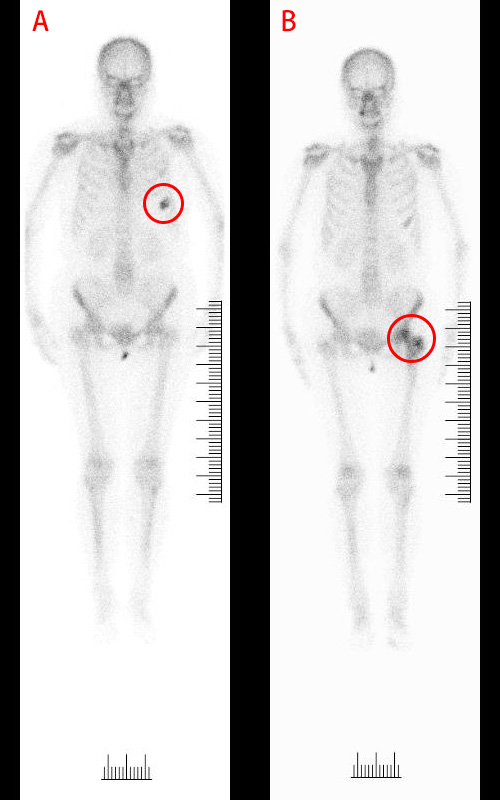

Supplement: Supplementary file 1 — Figure S1 Isotope bone scan at March 22th, 2016 (A) and March 21th, 2018 (B). [file TCA-12-2622-s001.jpg]
